# Design of the evolution of management strategies of heart failure patients with implantable defibrillators (EVOLVO) study to assess the ability of remote monitoring to treat and triage patients more effectively

**DOI:** 10.1186/1745-6215-10-42

**Published:** 2009-06-18

**Authors:** Maurizio Marzegalli, Maurizio Landolina, Maurizio Lunati, Giovanni B Perego, Alessia Pappone, Giuseppe Guenzati, Carlo Campana, Maria Frigerio, Gianfranco Parati, Antonio Curnis, Irene Colangelo, Sergio Valsecchi

**Affiliations:** 1 Cardiology Department, S. Carlo Borromeo Hospital, Milan, Italy; 2 Cardiology Department, Fondazione Policlinico S. Matteo IRCCS, Pavia, Italy; 3 Cardiovascular Department, Niguarda Hospital, Milan, Italy; 4 Cardiology Division, Istituto Auxologico S. Luca Hospital, Milan, Italy; 5 Cardiology Department, S. Raffaele University Hospital, Milan, Italy; 6 Cardiology Unit, Ospedali Civili, Brescia, Italy; 7 Clinical Department, Medtronic Italia, Rome, Italy

## Abstract

**Background:**

Heart failure patients with implantable defibrillators (ICD) frequently visit the clinic for routine device monitoring. Moreover, in the case of clinical events, such as ICD shocks or alert notifications for changes in cardiac status or safety issues, they often visit the emergency department or the clinic for an unscheduled visit. These planned and unplanned visits place a great burden on healthcare providers.

Internet-based remote device interrogation systems, which give physicians remote access to patients' data, are being proposed in order to reduce routine and interim visits and to detect and notify alert conditions earlier.

**Methods:**

The EVOLVO study is a prospective, randomized, parallel, unblinded, multicenter clinical trial designed to compare remote ICD management with the current standard of care, in order to assess its ability to treat and triage patients more effectively.

Two-hundred patients implanted with wireless-transmission-enabled ICD will be enrolled and randomized to receive either the Medtronic CareLink^® ^monitor for remote transmission or the conventional method of in-person evaluations. The purpose of this manuscript is to describe the design of the trial. The results, which are to be presented separately, will characterize healthcare utilizations as a result of ICD follow-up by means of remote monitoring instead of conventional in-person evaluations.

**Trial registration:**

ClinicalTrials.gov: NCT00873899

## Background

Hospitalization for heart failure (HF) is an increasingly serious clinical issue and carries a heavy economic burden [1]. Thus, new strategies to keep HF patients out of hospital are needed.

Based on positive outcomes from numerous randomized controlled trials, the current guidelines for the management of chronic HF [1] include the use of implantable defibrillators (ICD) and defibrillators for resynchronization therapy (CRT-D) as the standard care in selected chronic HF patients.

The ability of implantable devices to continuously monitor variables such as heart rate [2], the patient's daily activity [3], intra-thoracic impedance for the detection of fluid accumulation [4], the occurrence of arrhythmias [5], and the integrity of the system [6] may provide early warning of changes in cardiac status or safety issues and allow early management. A standard method of notifying alert conditions in ICD uses a programmable feature that enables a patient to be audibly alerted if any of the various parameters exceeds the pre-defined range [7].

ICD patients visit the clinic for routine device monitoring 2–4 times per year [8,9]. However, many of these routine device checks result in no programming or device changes [10], and many patients suffer no clinically significant events between visits. When these patients do have clinical events, such as ICD shocks or device alert notifications, they often visit the emergency department (ED) or clinic for an unscheduled examination. These unplanned visits place an even greater burden on healthcare providers.

Several major manufacturers of devices offer a technology for remote ICD monitoring [11-13], with the purpose of reducing unnecessary routine and interim visits, and allowing physicians to remotely access patients' data.

Earlier detection and notification, combined with remote management, may also have the effect of shifting healthcare visits from the ED to the clinic, thereby reducing costs and the burden on the healthcare system. In addition, remote monitoring with these enhanced features may improve patients' quality of life by reducing anxiety between follow-up visits.

This study has been designed to compare these remote management capabilities with the current standard of care, in order to assess their ability to treat and triage patients more effectively.

## Methods/design

### The system

The Medtronic CareLink^® ^system (Minneapolis, MN, USA) includes a patient monitor plugged into a standard analog telephone connection, and a lightweight wand to communicate with the implanted device [11]. Interrogation of the device and transmission of the data occur when the patient places the wand over the implanted device. Moreover, the system uses radiofrequency telemetry for automatic wireless communication. This allows data transmission without patient intervention and enables automatic transmission at scheduled intervals as well as alert-based downloads. Specifically, in the case of programmable parameters, the system can transmit data on diagnostic variables, arrhythmias, ICD therapies delivered and battery/lead issues, and can alert the physician via phone or e-mail.

The patient's information is sent to a secure Network server via the telephone connection. The clinical staff can review device information on a secure Website via the Internet. Available data are equivalent to those which can be retrieved at an in-office visit.

### Hypothesis

It is hypothesized that patients implanted with ICD/CRT-D devices endowed with the CareLink system will require less attention for cardiac or device-related episodes than patients whose devices do not have such features.

### Objectives

The primary objective of the study is to determine whether patients using the CareLink system display a different rate of unplanned cardiac or device-related in-hospital visits from patients in the standard arm. The endpoints for this objective include all cardiac and device-related clinic visits, i.e. all hospital admissions not involving an overnight stay, when the interval between the decision to admit and the admission is <24 hours. The events anticipated to prompt these visits are: ICD alerts regarding system integrity, atrial and ventricular arrhythmias, decrease in intra-thoracic impedance signifying possible fluid accumulation, and patients' symptoms. These endpoints will be subdivided according to whether the visits are related or not related to episodes of worsening of HF; estimation of their respective rates will constitute a secondary objective of the study.

Additionally, the rate and related costs of total healthcare utilizations (all planned and unplanned hospital admissions involving and not involving an overnight stay) for cardiac or device-related events will be compared between groups. For this objective, hospital visits will be also scrutinized and classified as necessary or unnecessary for the clinical management of the patient. In particular, each visit will be judged necessary if it results from an appropriate and clinically meaningful ICD alert (e.g.: intra-thoracic impedance alert associated with clinically deteriorated HF) and if the clinician was not previously aware of the clinical/ICD condition.

### Other variables

This study will also test whether the CareLink system:

• Reduces the time between the onset of asymptomatic events and the clinical decision on such events;

• Modifies the degree of patient anxiety, as measured by the composite scores of both the State and Trait components of the State-Trait Anxiety Inventory – Form Y;

• Modifies the patient's clinical status, as measured by the Clinical Composite Score [14];

• Modifies the patient's quality of life, as measured by the Minnesota Living with Heart Failure Questionnaire and the EQ-5D Questionnaire.

### Design

This is a one-to-one randomized, prospective study. The study has been approved by the Institutional Review Board of the 6 participating Italian centers.

**Figure 1:**
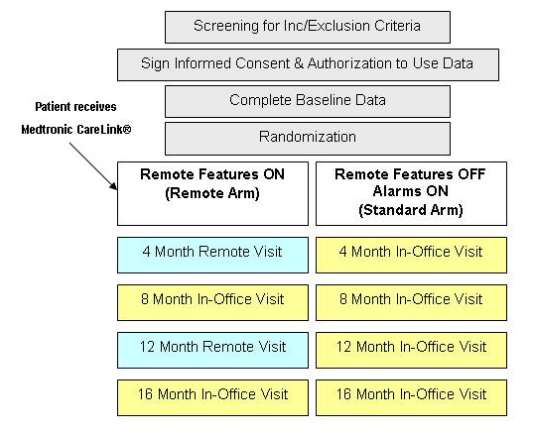
**Trial design**. Enrollment and follow-up schedule.

Patients implanted with wireless-transmission-enabled Medtronic ICD or CRT-D will be randomized to "remote transmission on" (remote arm) or "remote transmission off" (standard arm) (Figure 1).

Randomization will be stratified by center and by time from implantation (≤ 6 months and > 6 months) to minimize selection bias and preserve homogeneity between arms with regard to stability of clinical status.

In the remote arm, devices will be programmed to transmit over the CareLink Network. Devices in the standard arm will have all features available in their ICD/CRT-D, including the audible notification of alerts, but will not have access to the CareLink Network.

### Patient population

This study requires 200 patients that meet all inclusion criteria.

#### Inclusion criteria

• Left ventricular systolic dysfunction, left ventricular ejection fraction ≤35%, as documented at the moment of ICD implantation;

• Implantation with a wireless-transmission-enabled Medtronic ICD or CRT-D;

• Ability and willingness to undergo remote follow-up instead of scheduled routine in-office follow-up visits;

• Ability to attend all required follow-up examinations at the study center.

#### Exclusion criteria

• Age less than 18 years;

• Unwillingness or inability to give informed consent;

• Life expectancy less than 12 months;

• Participation in another clinical study that may have an impact on the endpoints of the present study.

### Procedures

Patients previously implanted with the above-mentioned devices will be brought in for a standard in-office examination and assessment of the inclusion criteria. After signing an informed consent form and being randomization to either the remote or standard arm, patients will be followed up for a 16-month period, with required visits after 4, 8, 12 months (only for standard arm) and 16 months. In the remote arm CareLink transmissions will replace the 4- and 12-month visits (Figure 1).

Table 1 summarizes the data to be collected at the baseline and during follow-up.

**Table 1:** Overview of data collection requirements

	**Baseline**	**4 Month**	**8 Month**	**12 Month**	**16 Month**
*Demographic/Medical History*	X				

*Device Information*	X				

*Randomization*	X				

*Medications*	X				X

*Healthcare Utilization*	X	X *	X	X *	X

*Alerts*	X	X	X	X	X

*Symptoms*	X	X *	X	X *	X

*Echocardiographic parameters*	X				X

*NYHA Class*	X	X *	X	X *	X

*Save-to-Disk ICD Data*	X	X *	X	X *	X

***Patient Questionnaires***					

*State Trait Anxiety Inventory*	X		X		X

*MLHF*	X				X

*EQ-5D*	X				X

*Patient and Caregiver Burden*	X				

* in Standard Arm only

### Device programming

Initial ICD programming is to be performed at the time of randomization. Tables 2 and 3 show detailed lists of all protocol-required device programming parameters.

**Table 2:** Alert programming. Clinical Management Alerts

	**Remote Arm**	**Standard Arm**
***Intra-thoracic Impedance Monitoring (OptiVol Alert)***		

*OptiVol Alert – Device*	Off	On

*OptiVol Alert – Monitor*	On	Off

***AT/AF Burden and Rate Settings***		

AT/AF Daily Burden Alert Enable – Device *	Off	On

AT/AF Daily Burden Alert Enable – Monitor *	On	Off

*- Daily AT/AF Alert Burden*	6 hours	6 hours

Avg. V. Rate During AT/AF Alert Enable – Device **	Off	On

Avg. V. Rate During AT/AF Alert Enable – Monitor **	On	Off

*- Avg. V. Rate During AT/AF*	100 beats/min	100 beats/min

*- Daily Burden for Avg. V. Rate*	6 hours	6 hours

***Number of Shocks Delivered during an Episode***		

*Alert Enable – Device*	Off	Off

*Alert Enable – Monitor*	On	Off

*- Threshold Number of Shocks*	1	-

***All Therapies in a Zone Exhausted for an Episode***		

*Alert Enable – Device*	Off	On

*Alert Enable – Monitor*	On	Off

* To pursue a "Rhythm Control" strategy; ** To pursue a "Rate Control" strategy

**Table 3:** Alert programming. Lead/Device Integrity Alerts

	**Remote Arm**	**Standard Arm**
*Patient Home Monitor*	On	Off

***Lead Impedance Out of Range*** (A. pacing, LV pacing*, RV pacing, RV defibrillation, SVC defibrillation**)		

*Alert Enable – Device*	On-High	On-High

*Alert Enable – Monitor*	On	Off

***Low Battery Voltage RRT***		

*Alert Enable – Device*	On-High	On-High

*Alert Enable – Monitor*	On	Off

***Excessive Charge Time EOS***		

*Alert Enable – Device*	On-High	On-High

*Alert Enable – Monitor*	On	Off

***VF Detection/Therapy Off***		

*Alert Enable – Device*	On-High	On-High

*Alert Enable – Monitor*	On	Off

* For systems with an LV lead; ** For systems with dual-coil leads

In summary, all alerts regarding clinical management (intra-thoracic impedance for fluid accumulation monitoring, atrial arrhythmias, ICD shocks delivered) will be turned on for wireless notification through the CareLink in the remote arm, while no audible alerts will be used. In the standard arm these alerts will be turned on for audible notification only.

All lead and device integrity alerts will be turned on for both wireless and audible notification in the remote arm, and for only audible notification in the standard arm.

In the standard arm, audible alerts for intra-thoracic impedance monitoring will be programmed to sound at a different time of the day from alerts for other causes, in order to distinguish them.

Clinicians are allowed to turn off the alert for atrial arrhythmias, if they are no longer attempting to control the subject's atrial rhythm. Similarly, they can turn off the alert for intra-thoracic impedance when recurrent inappropriate detections of fluid accumulation are documented.

### Management strategies

Clinics will be allowed to restrict notification of alerts to business hours, but will be encouraged to check the CareLink website at least once daily for transmissions.

**Figure 2:**
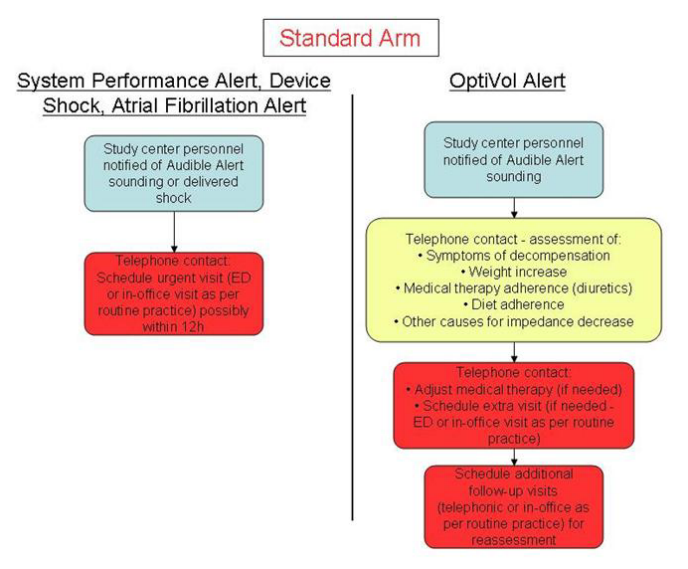
**Management strategy requirements**. Operative flowcharts for the management of device shocks and alerts in the standard arm.

**Figure 3:**
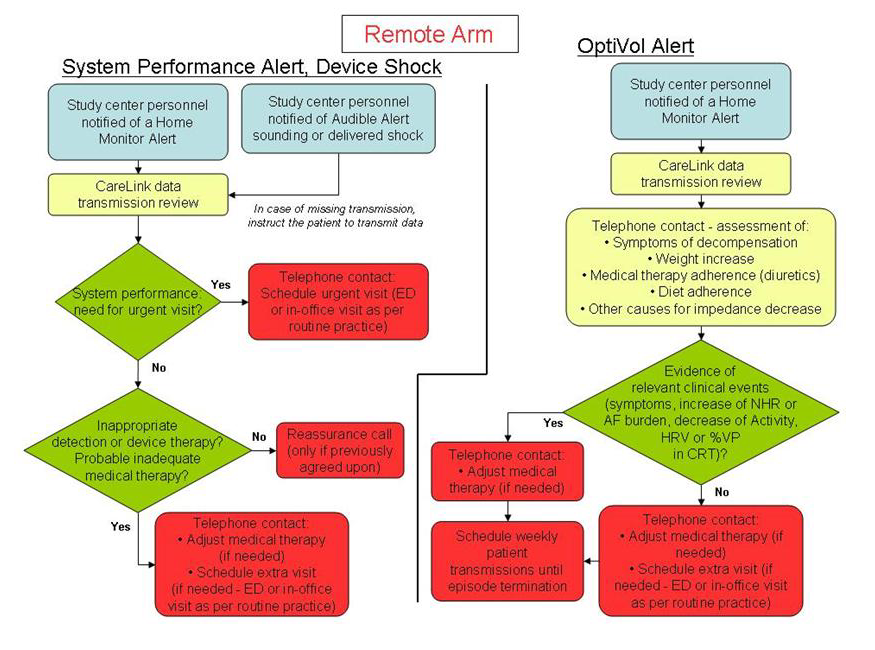
**Management strategy requirements**. Operative flowcharts for the management of system performance alerts, device shocks and OptiVol alerts in the remote arm.

**Figure 4:**
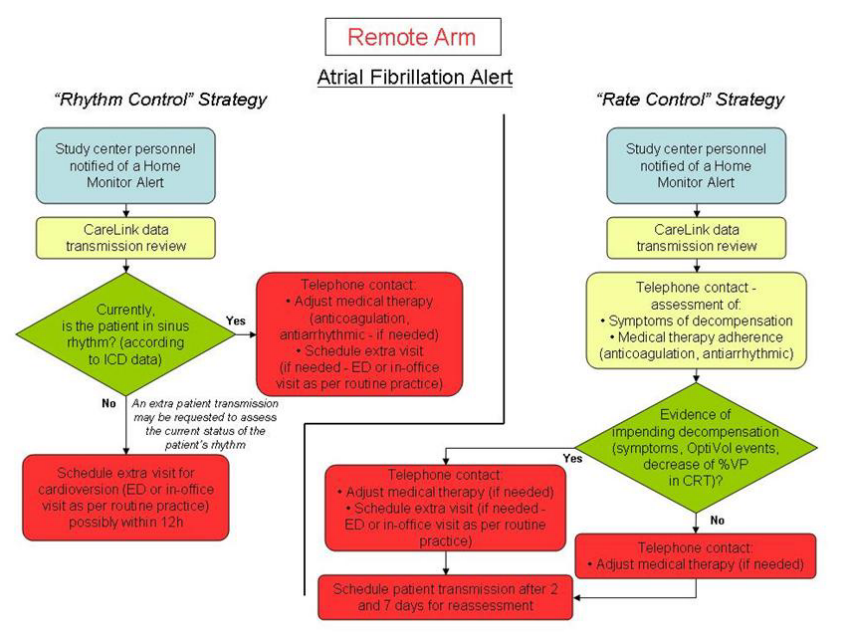
**Management strategy requirements**. Operative flowcharts for the management of atrial fibrillation alerts in the remote arm.

If the clinic is notified of an audible alert sounding (both arms) or an alert received via CareLink (remote arm only), or if subjects report signs or symptoms, the clinician is required to implement management strategies that exploit the respective features available in the two arms. A summary of management strategy requirements is listed in Figure 2 for the standard arm, and in Figure 3 and 4 for the remote arm.

Assessment of the patient's clinical status and verification of appropriate functioning of the ICD will be performed in accordance with the current guidelines and the center's routine practice.

### Statistical methods

An intention-to-treat analysis will be performed on all randomized subjects and will serve as the primary analysis for all objectives in this study.

The primary and secondary hypotheses will be tested by using the Comparison of Incidence Rates (Large Sample) Test. A two-sided test and a significance level of α = 0.05 will be used.

Cost analysis will be carried out from the perspectives of the healthcare system and the patient. Consequently, the unit costs considered will be related to the public tariffs or to the patient's out-of-pocket expenses. The in-hospital resources related to the introduction and utilization of CareLink will also be evaluated.

### Sample size justification

The sample size requirements for this study are intended to provide adequate power for the analysis of the primary objective.

Simulation methods used data extrapolated from a population of 160 CRT-D patients followed up for 224 ± 130 days (data on file). Overall, 220 episodes occurred over 98 person-years (2.2 per person-year):

• 140 intra-thoracic impedance alerts;

• 34 episodes of atrial arrhythmia >12 hours in a single day;

• 6 additional episodes of atrial arrhythmia >6 hours with ventricular rate >100 beats/min in a single day;

• 40 ICD shocks delivered.

The simulations were run in R Software 2.4.1 according to the following hypotheses: the incidence density used as a null hypothesis was 2.2 per person-year; this rate is expected to decrease by 10% in the study arm; it was assumed that 20% of patients would drop out uniformly over the follow-up period; the target type-I error (α) was 0.05.

A sample size of 100 subjects per group would allow a power of 99.8% (95% confidence interval: 96.0 – 100.0) to detect an incidence rate ratio of 0.90, with a type-I error of 0.05.

## Discussion

### Rationale for objectives

The CareLink system performs full interrogation and transmission of ICD data, and available information is no different from that retrieved during an in-office visit. The main advantage lies in the possibility of early reaction to patient symptoms and device alerts without the patient having to come to the hospital for either scheduled or unscheduled examinations.

As suggested [10,15], adoption of the CareLink system should enable cardiac or ICD-related episodes to be successfully managed through remote data review and telephone follow-up, without requiring additional in-hospital visits. This will be ascertained by measuring the reduction in the rate of unplanned in-hospital visits (primary objective). Moreover, it is hypothesized that this reduction will concern both visits related to the worsening of HF and those that are not HF-related, which in current clinical practice are managed by the heart failure specialist and the electrophysiologist, respectively. More generally, it is supposed that the rate and the costs of total healthcare utilizations for cardiac or ICD-related events will decrease; this will be tested as a secondary objective.

### Rationale for design

Currently, outpatient follow-up examinations of ICD patients are scheduled at regular 3- to 6-month intervals [8,9]. In the present study, we will compare a standard arm undergoing 4 in-office examinations per year with a remote arm in which 2 of the 4 examinations are replaced by remote interrogations, as previously proposed [16,17].

Acute events, such as ICD shocks, alerts or symptoms that usually prompt the subject to seek urgent attention at the clinic, will be managed via CareLink and telephone follow-up in the remote arm. Indeed, it has been recently shown that a considerable portion of post-shock interrogations do not involve reprogramming of the ICD and may therefore be performed remotely [10,15].

The audible alert feature has been shown to facilitate the early discovery of serious ICD complications [7], and improved alert algorithms have recently been implemented to detect lead failures promptly [6]. Similarly, in view of the high incidence of atrial fibrillation in HF [18] and the consequent increased risk of decompensation, stroke and inappropriate shocks, the advanced ICD capabilities for detecting atrial arrhythmias have the potential to play a growing role in patient monitoring [5]. By providing remote notification and access to details of the occurrence and duration of atrial fibrillation and ventricular response, CareLink will facilitate the management of therapies for rhythm or rate control and the prompt administration of anticoagulants for stroke prevention.

Continuous intra-thoracic impedance monitoring in modern ICD is intended to detect HF decompensation promptly, thereby permitting early therapeutic intervention [4]. Decreased intra-thoracic impedance has been shown to reliably identify acute HF, with low rates of undetected events [19], and to be associated with an increased risk of HF hospitalization [20]. As recently shown [15], a high percentage of episodes may be successfully and promptly managed through CareLink without requiring additional in-hospital visits, by means of remote data review and assessment of symptom status and therapy compliance by phone. Moreover, patients might not hear or respond to the audible impedance alert, as recently reported [21]. Thus, automated telemetric transmission of alerts to the physician may shorten the delay between alert and therapy initiation, further improving the clinical outcome.

Other trials have used a control arm blinded to device data [22], in order to test the clinical value of diagnostic features. By contrast, in the present study all alerts will be turned on for audible notification in the standard arm and the diagnostics will be open for in-office review. This will enable the benefits associated to remote notification and access to ICD data to be specifically assessed in addition to the diagnostic and alerting capabilities of modern ICD.

Another trial is currently investigating the CareLink system in pacemaker patients [23]. The present study on ICD/CRT-D patients, however, will focus on a sicker population with more complex devices. This is the setting in which remote monitoring is presumed to offer the greatest benefits.

In the hypothesis of a more stable clinical status of the patient and a reduced need to adjust ICD parameters 6 months after implantation [10], stratifying patient randomization on the basis of time since implantation will limit a potential bias.

### Summary

Through a retrospective analysis of device-stored data from patients implanted with CRT-D [10], we recently assessed the potential impact of remote follow-up in clinical practice and demonstrated that it can constitute a practical alternative to frequent scheduled and unscheduled in-office visits.

Subsequently, in the framework of the multicenter Italian CareLink evaluation, we showed that the ease of use, satisfaction and acceptance of the system in European clinical practice is high among both patients and clinicians [17]. We therefore suggested that, by improving the clinical management of tachyarrhythmias and HF episodes in CRT-D patients, this system may lead to a reduction in healthcare utilizations, if included in a disease management program [15]. This hypothesis is to be tested in the present randomized controlled study, which has been designed to compare the remote management of ICD/CRT-D patients with the current standard of care.

Patient enrollment began in May 2008 and is projected to end by May 2009. Follow-up will be for 16 months after the last enrollment and is expected to end in September 2010.

## Abbreviations

CRT-D: defibrillators for resynchronization therapy; ED: emergency department; ICD: implantable defibrillator.

## Competing interests

Dr. Marzegalli, Dr. Landolina, Dr. Lunati, Dr. Perego, Dr. Pappone, Dr. Guenzati, Dr. Campana, Dr. Frigerio, Dr. Parati, Dr. Curnis have declared no competing interests. Colangelo and Valsecchi are salaried Medtronic employees.

## Authors' contributions

MM is the Principle Investigator for the study described in the manuscript and made significant contributions to the study design and to drafting and revising the manuscript, and provided final approval of the version to be published. MLa participated in developing the concept of the study, revising the manuscript and reviewing the final version to be published. MLu participated in developing the concept of the study, revising the manuscript and reviewing the final version to be published. GBP participated in developing the concept of the study, revising the manuscript and reviewing the final version to be published. AP participated in developing the concept of the study concept, revising the manuscript and reviewing the final version to be published. GG participated in developing the concept of the study, revising the manuscript and reviewing the final version to be published. CC participated in developing the concept of the study, revising the manuscript and reviewing the final version to be published. MF participated in developing the concept of the study, revising the manuscript and reviewing the final version to be published. GP participated in revision of the manuscript and review prior to final approval of version to be published. AC participated in revision of the manuscript and review prior to final approval of version to be published. IC participated in developing the concept of the study, revising the manuscript and reviewing the final version to be published. SV participated in developing the concept of the study, revising the manuscript and reviewing the final version to be published.
